# Using the health belief model to predict breast self examination among Saudi women

**DOI:** 10.1186/s12889-015-2510-y

**Published:** 2015-11-23

**Authors:** Mostafa A. Abolfotouh, Ala’a A. BaniMustafa, Aisha A. Mahfouz, Mohammed H. Al-Assiri, Amal F. Al-Juhani, Ahmed S. Alaskar

**Affiliations:** King Abdullah International Medical Research Center (KAIMRC), Mail Code 1515, Riyadh, Saudi Arabia; King Saud bin-Abdulaziz University for Health Sciences (KSAU-HS), Riyadh, Saudi Arabia; King Abdulaziz Medical City, Ministry of National Guard - Health Affairs, POB 22490, Riyadh, 11426 Saudi Arabia

**Keywords:** Breast cancer, HBM, Saudi women, Breast self examination, Screening, Breast awareness

## Abstract

**Background:**

In the Kingdom of Saudi Arabia, breast cancer (BC) usually presents at advanced stages and more frequently in young pre-menopausal women in comparison to western countries. There is controversy surrounding the efficacy of breast self examination (BSE) for early detection of BC in countries where other methods are available. This study aims to explore the perception towards breast cancer and towards BSE among Saudi women, using the Health Belief Model (HBM).

**Methods:**

A convenient sample of adult Saudi female employees, working at King Abdulaziz Medical City, Riyadh, Saudi Arabia (*n =* 225), and their non-working adult female family members (*n =* 208), were subjected to the Arabic version of revised Champion’s Health Belief Model Scale (CHBMS) and the Arabic version of Breast Cancer Awareness Measure (CAM), to assess their knowledge and attitude on BC respectively. Percentage mean score (PMS) for each HBM domain was calculated. Significant predictors of BSE practice were identified using logistic regression analysis and significance was considered at *p <* 0.05.

**Results:**

The majority of women heard about BSE (91.2 %), only 41.6 % reported ever practicing BSE and 21 % performed it regularly. Reported reasons for not doing BSE were: not knowing how to examine their breast (54.9 %), or untrusting themselves able to do it (24.5 %). Women were less knowledgeable about BC in general, its risk factors, warning signs, nature and screening measures (PMS:54.2 %, 44.5 %, 61.4 %, 53.2 %, 57.6 % respectively). They reported low scores of; perceived susceptibility, seriousness, confidence and barriers (PMS: 44.8 %, 55.6 %, 56.5 % & 41.7 % respectively), and high scores of perceived benefits and motivation (PMS: 73 % & 73.2 % respectively) to perform BSE. Significant predictors of  BSE performance were: levels of perceived barriers (*p =* 0.046) and perceived confidence (*p =* 0.001) to BSE, overall knowledge on BC (*p <* 0.001), work status (*p =* 0.032) and family history of BC (*p =* 0.011).

**Conclusions:**

Saudi women had poor knowledge on BC, reported negative attitude towards BSE and their practice was poor. Working women and those with family history of BC, higher perceived confidence and lower perceived barriers on HBM, and those with high level of knowledge on BC were more likely to perform BSE. Breast awareness as an alternative to BSE needs further investigations. HBM was shown as a valid tool to predict BSE practice among Saudi women.

## Background

Breast cancer is the most frequent malignancy of women worldwide. It is the leading cause of female cancer related disability and mortality. in Saudi Arabia, breast cancer is the most common type of cancers among Saudi females and accounted for more than 25 % of all newly diagnosed cancer among them [1], with the proportion of young age-onset much higher than in western countries [2, 3]. It is usually present at advanced stages [4]. Many women miss early detection and treatment opportunities owing to lack of information, knowledge and awareness of breast cancer as well as to cancer screening practices [5].

A significant number of women present with advanced stages of the disease due to lack of information, knowledge and awareness of early detection measures. Previous studies showed limited knowledge about breast cancer screening, and few women performed screening for early detection purposes [6–8]. A recent study reported very low rates of breast cancer screening in Saudi Arabia, a country with free health services, and educational campaigns were recommended to improve BC screening and to address the barriers for BC screening [9].

Breast self-exam (BSE), mammography and clinical breast examination (CBE) are considered as screening methods for early detection of breast cancer. There is controversy surrounding the efficacy of BSE in countries where mammography and clinical breast exams are readily available. Data from two large trials in China and Russia [10–12] did not suggest a beneficial effect of screening by BSE, but reported increased harm in terms of increased number of benign lesions identified and an increased number of biopsies performed. However, in 2009, the U.S. Preventive Services Task Force concluded that there is insufficient evidence to recommend for or against teaching or performing routine breast self-examination [13]. The American Cancer Society now recommends the pros and cons of BSE be reviewed with women beginning in their 20s and that the ultimate decision of whether to practice BSE be left up to the individual [14].

In the absence of evidence that routine, systematic BSE reduces deaths from BC, a number of international organizations recommended that women look and feel for breast changes as part of general body awareness, while dressing or showering, so as to be aware of any changes from what is normal for them. This concept is known as “breast awareness (BA)” [15]. It is possible that increased breast awareness may have contributed in the decrease in mortality from breast cancer in some countries, although uncertainty exists whether the benefits of BA outweigh the harms [16].

Most women in previous studies held pessimistic views about the curability of breast cancer (58.2 %) [17]. The Health Belief Model (HBM) has been used in several studies as a theoretical framework to study BSE and other breast cancer detection behaviors [18]. The HBM has been translated, tested and used for women of different cultures. The model stipulates that health-related behavior is influenced by a person’s perception of the threat posed by a health problem and by the value associated with his/her action to reduce that threat [19]. According to the HBM scale, a woman who perceives that she is susceptible to breast cancer and that breast cancer is a serious disease would be more likely to perform regular breast examinations. Similarly, a woman who perceives more benefits of and fewer barriers to BSE would be more likely to practice BSE [20]. In previous studies, BSE screening was linked to perception of risk [21, 22], perceived benefits [18, 22, 23], barriers [18, 22–24], confidence [18, 21, 23, 24], having heard/read about BC [24], motivation 18], susceptibility [18, 23], Knowledge of BSE issues [21, 23], employment [21], demographic characteristics [23] and regular visits to a physician [23, 24].

In Saudi Arabia, although some studies were conducted on BSE [17, 25, 26], yet none of these studies investigated women’s perception using the HBM. This study was designed to study the perception towards breast cancer and BSE among a group of Saudi women, using the HBM, through the following: 1) assessment of women’s beliefs and attitudes surrounding breast cancer and breast self examination using the health belief model, 2) determination of the level of knowledge among Saudi women regarding breast cancer and breast cancer-related practices, 3) determination of breast cancer-related behaviors, and 4) identification of factors influencing the practice of BSE. The results of this study may provide a baseline assessment for future intervention programs to promote early detection and early management of BC.

## Methods

### Study area/setting

Study was conducted in outpatient clinic at King Abdulaziz Medical City (KAMC), Riyadh , Saudi Arabia. KAMC commenced its operations in may 1983. Since then it has continued expanding while providing service for a rapidly growing patient population in all of its catchments areas. At KAMC, the bed capacity increased to 1000 beds in addition to 25 beds allocated for expected surgical operations in Ward 19, and 132 beds for admission of emergency cases. The healthcare quality on an international scope, NGHA has passed the requirements for accreditation under the Joint Commission International (JCI) standards with excellent performance in December, 2006.

### Study subjects

Saudi female employees above 18 years old, working at King Abdulaziz Medical City, (KAMC), Riyadh, Saudi Arabia, and their non-working adult female family members. , who were willing to participate, made the target of the study..In Saudi culture, paternalistic approach is practiced when dealing with females, that would not allow an easy access to non-working females to participate in the study. Thus, each working woman was asked to invite her non-working adult female family member(s) to participate in the study by filling the questionnaire. Thus, work status as a possible predictor of women’s perception and practice on BSE, was investigated. Doctors and nurses were excluded so as to allow for the study be representative of Saudi women in general.

### Study design

A cross sectional study was applied using self-adminestered questionnaire.

### Sample size and sampling technique

Based on the assumption of 58.2 % of women holding pessimistic views about the curability of breast cancer in a previous study [17], and with a 5 % margin of error and 95 % CI, the estimated sample size was 374 women. This sample was allocated equally from the working and non-working females. Working women were selected by convenient sampling from adult female employees at KAMC, Riyadh, Saudi Arabia during the time of study (*n =* 225), Each female employee who showed interest to participate in the study was asked to take a questionnaire home to be filled by one non-working adult female family member, and to return the filled questionnaire the day after. Thus, a total of 433 females (225 employees & 208 non-working females) participated in the study.

### Data collection

The questionnaire was initially designed based on previously validated questionnaires [4, 17, 27–29]. It is originally designed in English and then translated into Arabic language and was validated in a pilot study before finally utilized. The questionnaire is composed of five sections:

1. Section one was to collect data on demographical characteristics of participants;
2. Section two was about knowledge of participants about breast self-examination;
3. Section three was about level of practice of breast self-examination;
4. Section four was the use of Breast Cancer Awareness Measure (Breast CAM) version 2 [30] to collect data from Saudi females about knowledge of participants regarding screening tests, nature of breast cancer, warning signs of breast cancer and risk factors. To assess reliability of the tool, test-retest reliability was done on a pilot sample of 20 women (10 working & 10 non-working) and Cronbach’s alpha was 0.82; and
5. Section five was the use of the health belief model (HBM) to collect data about women’s perception to BSE. The Arabic version of revised Champion’s Health Belief Model Scale (CHBMS) [31] was tested for validity and reliability in Mikhail and Petro-Nustas [20] and found satisfactory. It consists of 6 concepts: perceived susceptibility to illness (5 items), perceived seriousness of illness (7 items), perceived benefits for the presumed action (6 items), perceived barriers for the presumed action (7 items), confidence in one’s ability (11 items) and health motivation (7 items) [32]. All the items have 5 response choices ranging from strong disagreement (1 point) to strong agreement (5 points). All scales are positively related to screening behavior, except for barriers which are negatively associated. Reported Cronbach’s alpha for the CHBMS ranged from 0.69 to 0.83. The reliability of these subscales for this study ranged from 0.78 to 0.89. The CHBMS was used after securing written permission from the author. The tool was pilot tested, few revisions were made and then administered as a self administrative questionnaire.

### Ethical considerations

Participation in the study was voluntary, and each participant was able to withdraw from the study at any time. The investigators explained the aim of the study to the participants. Agreement to fill the questionnaire was considered as a consent to participate in the study. The study protocol received ethical approval from the IRB of the Saudi National Guard Health Affairs, Riyadh, Saudi Arabia, (*application number SP14/107*).

### Data management

Data entry and statistical analysis were performed using SPSS® version 20.0 (IBM Corporation, Armonk, NY, USA). Descriptive statistics, such as percentages, frequencies, means, and standard deviations, were used to measure the demographic variables and the responses to knowledge and attitude statements. Analytical statistics were applied to investigate the association of knowledge and attitude with demographic variables. Logistic regression analysis was performed to identify the significant predictors for BSE practice. Statistical significance was set at *p <* 0.05 for all analyses.

## Results

Table 1 shows the distribution of 433 women according to some socio-demographic characteristics. The majority of women were of age 18 to less than 35 years (62.1 %), with an average age of 39.4 ± 7.2 years. The majority were secondary or more educated ( 95.6 %), one-half were employees (52 %), and one-half from higher income families of more than 10,000 Saudi Riyals (53.8 %). Family history of BC was reported by 22.9 % of women (3.3 % near relatives & 19.6 % far relatives).

**Table 1:** Women’s Sociodemographic characteristics

Demographic characteristics	no (*N =* 433)	%
*Age in years:*		
18 - < 35	294	68.1
35 – 45	86	19.9
<45	52	12.0
(Mean ± SD)	39.4 ± 7.2	
*Education level:*		
< Secondary	19	04.4
≥ Secondary	414	95.6
*Marital status:*		
Single	206	47.6
Married	196	45.2
Widow/Divorced	31	07.2
*Work status:*		
Employee	225	52.0
Non-employee	208	48.0
*Monthly income:*		
<5000 SR	62	14.3
5000-10000 SR	138	31.9
>10000 SR	233	53.8
*Family history of BC:*		
No	334	77.1
Near relatives (Mother/Sister/Daughter)	14	03.3
Far relatives	85	19.6

*SR* Saudi riyal, *BC* breast cancer

Table 2 shows the distribution of women according to knowledge and practice of BC and BSE issues. Almost all women (91.2 %) in the present study heard about BSE, mostly from educational public campaigns (54.7 %), TV (40.5 %) and internet (38.2 %). With regard to knowledge of BSE, about three-quarters of all women (74.7 %) reported the of ≥19 years as the age of initiation of BSE, and only 43.5 % reported that it should be done monthly, and 44.8 % reported that it should be done five days after menses, respectively.

**Table 2:** Women’s Knowledge and reported practices on BSE

(A) Knowledge about BSE	no (*N =* 433)	%
*Have you heard about BSE*		
Yes	395	91.2
No	38	08.8
*Age of SBE starting (n = 395)*		
<19	17	04.3
≥19	295	74.7
Don’t know	83	21.0
*How often could BSE be done per year (n = 395* **)**		
Daily	1	0.3
Weekly	3	0.8
Monthly	172	43.5
Once per year	150	37.9
Don’t know	69	17.5
*When BSE should be performed with regular menses (n = 395)*		
Regular day of each month	31	07.9
5 days after	177	44.8
Do not know	187	47.3
*Source of information (n = 395)* ^*a*^		
TV	160	40.5
Radio	16	04.1
Educational camping Public	216	54.7
Internet	151	38.2
Medical journal	70	17.7
Primary health care	50	12.7
Friends	39	09.9
Other	37	09.4
(B) BSE Practices	no (*N =* 433)	%
*Have you done BSE before (n = 433)*		
Yes	180	41.6
No	253	58.4
Number per year (Mean ± SD)	4.1 ± 6.3	
*Where do you perform BSE (n = 181)* ^*a*^		
Home	134	74.0
Early detection programs	7	03.9
Private hospital	22	12.2
Governmental hospital	33	18.2
*The last time performed BSE (n = 180)*		
Less than 1 month	38	21.1
Less than 1 year	82	45.6
More than 1 year	60	33.3

^*a*^Non mutually exclusive

With regard to practice of BSE, less than one-half (41.6 %) of women reported that they have practiced BSE for 1 to 12 times with an average of 4.1 ± 6.3 times per year. The average age of initiation of BSE was 26.8 ± 7.7 years. Only 21.1 % reported performing it in less than a month, 45.6 % in less than a year, while 33.3 % reported not doing it for more than a year. Home was the main place for the majority of women where to do BSE (74.0 %), followed by hospitals (30.4 %).

**Fig. 1:**
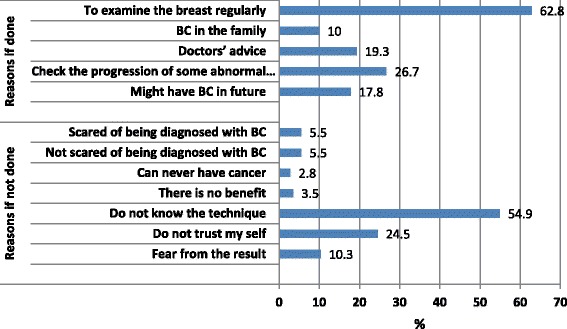
Reasons of doing and not doing BSE as reported by women

Reasons for doing BSE as reported by 180 women were: to examine their breast regularly (62.8 %), and to check for the progression of some abnormal changes (26.7 %), or because of doctors’ advice (19.3 %). Reasons for not doing BSE as reported by 235 women were: because of not knowing how to examine their breast (54.9 %), or because of untrusting themselves able to do it (24.5 %),  Fig. 1.

### Knowledge about BC

**Fig. 2:**
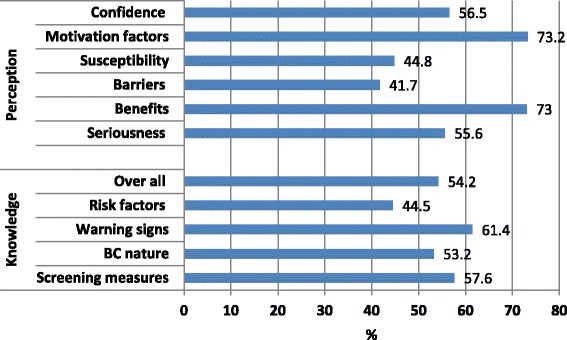
Percentage mean score of knowledge and attitude scales

The overall percentage mean score (PMS) of knowledge is (54.2 %), denoting low level of knowledge. This PMS was the lowest for the knowledge of risk factors (44.5 %), and the highest for the knowledge of warning signs (61.4 %),  Fig. 2.

With regard to knowledge of screening measures, about one-half of women reported knowing how to perform BSE, and less than a half reported knowing mammography (41.1 %), and clinical breast examination (44.2 %), with an overall PMS of 57.6 %. Regarding the knowledge of breast cancer (BC), the majority of women (88.2 %) reported that BC is curable in its early stages, and less than two-thirds (60.5 %) considered it as fatal if not treated, common in women over 50 (61.4 %), and that nipple discharge is important (60.3 %). Only 22.6 % and 18.1 % reported that it is common in obese, and can be in both breasts, respectively,  Table 3

**Table 3:** Women’s responses to different statement on breast cancer issues

Knowledge domains	Yes		No		Don’t know	
	no	%	no	%	no	%
Screening						
Do you know about mammogram	178	41.1	230	53.1	25	05.8
Do you know how to perform BSE	231	53.3	176	40.7	26	06.0
Do you know about clinical examination of breast	191	44.2	205	47.5	36	08.3
Is it possible for screening measures to enhance the chance of recovery	396	91.4	15	03.5	22	05.1
Breast Cancer						
BC is curable in early stages	382	88.2	5	01.2	46	10.6
BC is highly mortality without treatment	262	60.5	45	10.4	126	29.1
Painless in early stages	215	49.7	36	08.3	182	42.0
BC more common in women over 50	266	61.4	42	09.7	125	28.9
Occurs in one breast only	164	38.2	75	17.5	190	44.3
BC more common in obese women	98	22.6	98	22.6	237	54.8
Warning signs						
Nipple discharge is important	261	60.3	37	08.5	135	31.2
A lump is definitely cancer	63	14.6	278	64.4	91	21.0
Breast lump	324	74.8	32	07.4	77	17.8
Early menarche	102	23.6	114	26.4	216	50.0
Sudden and abnormal changes in size	319	73.7	21	04.8	93	21.5
Discharges from nipple	292	67.4	31	07.2	110	25.4
Changes in nipple shape	292	67.6	25	05.8	115	26.6
Risk Factors						
Radiotherapy	149	34.5	56	13.0	227	52.5
Hormonal replacement	135	31.3	51	11.8	246	56.9
Obesity	121	28.0	114	26.4	197	45.6
Practice physical exercise	39	09.0	293	67.8	100	23.2
Smoking	232	53.6	61	14.1	140	32.3
Alcohol	220	50.8	54	12.5	159	36.7
Increase with age	193	44.6	91	21.0	149	34.4
Low fat intake	45	10.4	200	46.2	188	43.4
Late menopause	69	15.9	116	26.8	248	57.3
Long oral contraceptive pills	158	36.5	70	16.2	205	47.3
Family history of breast cancer	307	71.2	39	9.1	85	19.7
Breast feeding practice	20	04.6	318	73.5	95	21.9
Trauma to breast area	149	34.4	95	21.9	189	43.7

.

The majority of women reported the breast lump (74.8 %) and the abnormal changes in breast size (73.7 %) as warning signs, and two-thirds reported discharge from the nipple (76.4 %) or change in its shape (67.6 %). Early menarche as a warning sign was reported by only 23.6 % of all women, Table 3.

As for knowledge of risk factors of BC, low proportions of women reported correctly the different risk factors, that varied from 15 % for late menopause, 28 % for obesity, 31.3 % for hormone replacement therapy (HRT), 36.5 % for oral contraceptive pills (OCP), 44.6 % for increased age to 50.8 % and 53.6 % for alcohol and smoking respectively. However, the majority of women reported that neither breast feeding practice (73.4 %) nor physical exercising (67.8 %) is a risk factor, while only 21.9 % of women reported that trauma to the breast is not a risk factor for BC, Table 3.

### Perception towards BSE

Figure 2 shows the PMS of different domains of attitude to BC and BSE as measured by the health belief model (HBM). The overall PMS of seriousness domain is low (55.6 %.), with less likelihood to practice BSE. However, the majority of women disagreed/strongly disagreed that BC is hopeless disease (78.5 %) and that they would not live more than 5 years with BC (66.2 %). Also, the overall PMS for susceptibility domain is low (44.8 %), with less likelihood to practice BSE. Only less than 10 % of all women who agreed/strongly agreed they; are susceptible to BC in the future (6.7 %), feel susceptible to BC (7.6 %), feel susceptible than anyone (4.8 %), feel the chance of getting BC as big (4.1 %), or feel highly susceptible to BC in the next 10 years (3.5 %), Table 4.

**Table 4:** Women’s responses to the different attitude items of the HBM

Attitude	Disagree/ Strongly disagree		Neutral		Agree/ Strongly agree	
	no	%	no	%	no	%
Seriousness						
BC is a hopeless disease	340	78.5	71	16.4	22	5.1
I think I will not live more than 5 ys. with BC	286	66.2	108	25	38	8.8
When I think about BC my heart beat faster	228	52.7	77	17.8	128	29.5
I am afraid even to think about BC	215	49.7	55	12.7	163	37.6
If I got BC this will threaten my marital life	166	38.3	138	31.9	128	29.8
All my life will be changed if I got BC	160	37.0	124	28.7	148	34.3
I think the problem about BC will persist long	98	22.6	152	35.1	183	42.3
The thought of BC scare me	81	18.7	47	10.9	305	70.4
Susceptibility						
I am susceptible to breast cancer in the future	188	43.4	216	49.9	29	6.7
I feel that I am susceptible to breast cancer	242	55.9	158	36.5	33	7.6
I think I am susceptible to breast cancer more than anyone	280	64.7	132	30.5	21	4.8
My personal chance of getting breast cancer is big	253	58.6	161	37.3	18	4.1
I am highly susceptible to breast cancer next 10 years	231	53.3	187	43.2	15	3.5
Confidence						
I know how to perform BSE	134	30.9	145	33.5	154	35.6
I am confident in performing BSE correctly	117	27.0	183	42.3	133	30.7
I am sure of the steps of BSE	152	35.1	170	39.3	111	25.6
I can use the correct parts of my fingers when performing BSE	139	32.1	168	28.8	126	29.1
I am confident I can discover breast tumors by performing BSE	152	35.1	200	46.2	81	18.7
I can discover breast tumor at size of big spot	90	20.8	148	34.2	195	45.0
I can discover breast tumor at size of small peas	172	39.8	181	41.8	80	18.4
I am able to discover breast tumors alone through performing BSE	178	41.1	193	44.6	62	14.3
I can discover breast tumor at size of small spot	188	43.4	197	45.5	48	11.1
I am able to differentiate between normal and abnormal breast tissue through BSE	196	45.3	171	39.5	66	15.2
When I look at mirror I can identify abnormal changes in my breast	127	29.4	130	30.0	176	40.6
Benefits						
Performing BSE monthly help in early detection of BC	26	6.0	67	15.5	340	78.5
Performing BSE monthly help in detection of tumors before going to doctors	40	9.2	94	21.7	299	69.1
Performing BSE monthly will decrease complications of BC if I got it	38	8.8	117	27.1	277	64.1
Performing BSE decrease the chance of making operation if I got it	52	12.0	147	33.0	234	54.0
When I performed BSE I became self-satisfied	57	13.2	155	35.9	220	50.9
Performing BSE decrease the anxiety about BC	44	10.2	116	26.8	273	63.0
Barriers						
Performing BSE is a trivial thing	370	85.6	42	09.7	20	4.7
Performing BSE is unfavorable thing	276	63.7	94	21.7	63	14.6
No private place at home to perform BSE	349	80.7	57	13.2	26	6.1
Feeling of shame and embarrassment when performing BSE	310	71.6	70	16.2	59	12.2
Performing BSE takes long time	239	55.2	162	37.4	32	7.4
Performing BSE increase my anxiety about liability of having BC	244	56.3	95	22.0	94	21.7
I think getting breast cancer is a destiny and BSE will not change it	336	77.6	68	15.7	29	6.7
Motivation factors						
Keeping my good health is important to me	5	1.2	15	03.5	413	95.3
I wish to discover health problems that occur early	9	2.1	15	03.5	409	94.4
I always seek new information that improve my health	19	4.4	49	11.3	365	84.3
I feel the importance of activities that improve my health	44	10.1	49	11.3	340	78.6
My diet contains complete and balanced meals	116	26.8	133	30.7	184	42.5
I practice exercise at least 3 times weekly	180	41.5	93	21.5	106	37.0
I perform periodic medical checkup	204	47.1	70	16.2	159	36.7

.

The overall PMS of confidence domain is low (56.5 %), reflecting less likelihood to practice BSE. About one-third of all women agreed/strongly agreed that they know how to perform BSE (35.6 %), and that are confident in performing BSE correctly (30.7 %), they are sure of the steps of BSE (25.7 %), and can use the correct parts of their fingers when performing BSE (29.1 %). However, only 18.7 % reported being confident to discover breast tumors by performing BSE. The ability to discover breast tumor at different sizes was agreed/strongly agreed by 45.0 %, 18.4 % and 10.0 % of women, for 3 sizes of lumps respectively, Table 4.

On the other hand, the overall PMS of benefit domain is reasonable (73.0 %), with more likelihood to practice BSE. However, the majority of women agreed/ strongly agreed that performing BSE monthly would help in; early detection of BC (78.5 %), detection of tumors before going to doctors (69.1 %), decreasing complications and chance of operation (64.1 % & 54.0 %, respectively), if they got BC. The overall PMS of barrier domain is low (41.7 %), with more likelihood to practice BSE. The majority of women disagreed/strongly disagreed that performing BSE; is a trivial thing (85.6 %), has no private place to do (80.7 %), would not change the fact that getting BC is a destiny (77.6 %), would make shame and embarrassment (71.6 %), or is unfavorable thing (63.7 %), Table 4.

The overall PMS of motivation factors domain is reasonable (73.2 %), reflecting more likelihood to practice BSE. The majority of women agreed/strongly agreed that; keeping their good health is important to them (95.3 %), they wish to discover health problems early (94.4 %), they always seek new information, and feel the importance of activities, that improve their health (84.3 %78.6 % respectively). However, small proportion of women agreed/strongly agreed that; their diet contains complete and balanced diet (42.5 %), they practice exercise at least 3 times weekly (37.0 %), or they perform periodic medical checkup (36.7 %), Table 4.

**Fig. 3:**
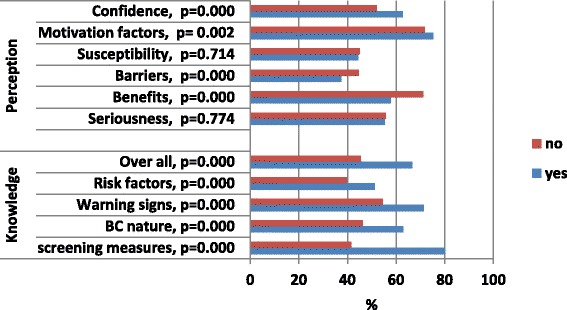
Relationship between BSE performance (Yes & no) and percentage mean scores of knowledge and attitude scales

Figure 3. shows that the PMS of overall knowledge of BC issues was significantly higher among women who practiced BSE than among those who have not (66.7 % versus 45.5 %, t = 11.78, *p <* 0.001). Women who practiced BSE showed significantly higher in all domains of knowledge: knowledge of screening measures (80.2 % versus 41.6 %, t = 16.59, *p <* 0.001), of BC nature (62.0 % versus 46.2 %, t = 8.13, *p <* 0.001), of warning signs (71.3 % versus 54.4 %, t = 6.15, *p <* 0.001), and of risk factors (51.3 % versus 39.7 %, t = 5.30, *p <* 0.001).

Figure 3 shows that women who practiced BSE had significantly higher benefits (75.8 % versus 71.1 %, t = 3.69, *p <* 0.001) and motivation factors scores (75.2 % versus 71.7 %, t = 3.15, *p =* 0.002), and lower barrier scores (37.5 % versus 44.6 %, t = 5.82, *p <* 0.001). than those who did not. However, both groups of women were comparable in seriousness and susceptibility PMS.

Table 5 shows the logistic regression analysis of BSE practice among women, with some independent variables. After adjustment for all possible confounders, it was found that the significant predictors to practice BSE were: positive family history of BC (*p =* 0.011), employment (*p =* 0.032), higher PMS of overall knowledge of BC issues (*p <* 0.001) higher PMS of confidence domain (*p =* 0.001) and lower PMS of barriers domain (*p =* 0.046).

**Table 5:** Logistic regression analysis of BSE performance with some independent variables

Independent predictors	*B*	*S.E*.	p-value	OR	95 % C.I
Age group	-.231	.305	.448	.794	.437: 1.442
Educational status	1.549	.908	.088	4.707	.794:27.894
Marital status	.441	.273	.106	1.554	.910:2.654
Monthly income	-.110	.262	.675	.896	.536:1.497
Knowledge score	.054	.008	. < 0.001^a^	1.055	1.039:1.072
Seriousness score	.013	.010	.186	1.014	.994:1.034
Benefits score	.010	.010	.352	1.010	.989:1.030
Barriers score	-.025	.012	.046^a^	.976	.952:1.000
Susceptibility score	.010	.008	.208	1.011	.994:1.027
Motivation score	.001	.012	.920	1.001	.978:1.024
Confidence score	.032	.009	.001^a^	1.032	1.013:1.052
BC Family history	.744	.292	.011^a^	2.104	1.188:3.726
Employment status	.561	.261	.032^a^	1.752	1.051:2.921
Constant	−8.251	1.732	<.001	<.001	

*CI* Confidence intervals

^a^Statistically significant

## Discussion

This study focused on defining the health beliefs of Saudi women regarding BSE and the influencing factors. The literature supports the argument that regular practice of BSE influences treatment, prognosis and survival rates [33, 34]. In the present study, almost all women heard about BSE. This figure was higher than other figures in the previous studies in Saudi Arabia such as; Jeddah (39.6 %) [35], Qassim (12 %) [36] and Riyadh (10.4 %) [37]. However, it was comparable with figures in other countries such as Sweden [38], Austria [39], Nigeria [40] and Egypt (10.4 %) [41].

In our study, less than one-half (41.6 %) of women reported that they have practiced BSE for 1 to 12 times with an average of 4.1 ± 6.3 times per year. The average age of initiation of BSE was 26.8 ± 7.7 years. Only 21.1 % reported performing it in less than a month, 45.6 % in less than a year, while 33.3 % reported not doing it for more than a year. This figure is comparable with that reported in studies conducted in Europe (44 %) [42], Hong Kong (52 %) [43], Italy (30 %) [44], Nigeria (11 %) [45] and Malaysia (19 %) [46], and is higher than figures reported in studies in Alexandria, Egypt (2.65 %) [41], Tehran, Iran (6 %) [47], Qassim, KSA (19 %) [36] and Istanbul, Turkey (10.2 %) [48]. Home was the main place for the majority of women where to do BSE (74.0 %), followed by hospitals (30.4 %).

In the literature, the main reasons for not-practicing BSE among those who claimed not to practice BSE included being not informed about how to practice it [49], fear or anxiety to discover the presence of a serious disease [27, 50], or not fully convinced regarding the importance of BSE [27, 50]. In the present study, reasons for not doing BSE as reported by 235 women were: because of not knowing how to examine their breast, or because of untrusting themselves able to do it. This finding justify why confidence in performing BSE in the HBM was a significant predictor of BSE practice. Being breast aware should be emphasized more than being BSE technique –abled [16].

According to the HBM, a woman who perceives that she is susceptible to breast cancer and that breast cancer is a serious disease would be more likely to perform regular breast examinations. Similarly, a woman who perceives more benefits of and fewer barriers to BSE would be more likely to practice BSE [20]. In the present study, women reported lower scores of susceptibility, seriousness and confidence that are in favour of not performing BSE. However, the higher benefits, motivation and confidence levels and lower barrier levels are in favour of doing BSE. BSE performance was directly associated with higher scores of confidence, motivation and confidence to perform BSE, and indirectly associated with barriers to perform BSE. However, after adjusting for sociodemographic characteristics, family history of BC and total knowledge score of BC, barriers and confidence domains of HBM were the significant predictors of BSE performance. Other significant predictors of BSE performance were higher scores of the overall knowledge on BC, employment status and positive family history of BC. These findings were in agreement with the results of previous studies [21–23], and could reflect the validity of use of the HBM in prediction of BSE performance among Saudi females.

The findings of the present study indicate that women with higher levels of BSE confidence have lower risks for not doing BSE. This was in agreement with previous studies [50]. Furthermore, women having lower levels of BSE barriers have higher potential for doing BSE. Results of previous research; barriers, health motivation, BSE benefits, and susceptibility are all related to BSE behavior [51, 52]. These results complied with the structure of HBM. On the basis of HBM theory, high perceptions of health motivation, BSE benefits, BSE self-efficacy and low perceptions of barrier and perceived susceptibility to breast cancer demonstrate increased levels of BSE status [18, 53, 54].

The socio-demographic characteristics of individuals can directly influence their attitude and indirectly affect health-related behavior [51]. Previous researches revealed that the health motivation of the participants who were well educated is quite high than who were not [48, 55–57]. In the present study, after adjustment for other potential confounders, education level was not a significant predictor of BSE performance. This was in agreement with previous studies [28], however it was not in agreement with the results of other studies that emphasized the relationship between the women’s educational status and BSE performance [18, 58]. In our study, the majority of women were educated, with only a few of them who were non-educated. The role of age in the frequency of BSE practice is controversial, while some study showed a negative association between age and BSE; others reported a positive association [28]. However, in the present study, age was not a significant predictor of BSE practice. This was also the situation for marital status, and monthly income.

It has been reported that the multi-responsibilities of working women, and shortage of time urge the working women to postpone their own affairs for the sake of other family members [27]. However, in the present study, employment was the only significant socio-demographic predictor of BSE practice, and working women were more likely to perform BSE Working women are more exposed to the different sources of information about BC and more likely to be enrolled in educational campaigns that were shown as the main source of information in the present study.

Adequate accumulation of knowledge on breast cancer has a positive effect on BSE practice [59, 60]. A significant number of women present with advanced stages of the disease due to lack of information, knowledge and awareness of early detection measures [28]. In the present study, lower levels of knowledge on BSE in particular and on BC in general were detected. The overall level of knowledge on BC was low. Less than one-half of all women reported correct information about the timing or frequency of BSE performance. Knowledge of screening measures was also low. All domains of knowledge on BC were significantly associated with BSE performance. Meanwhile, the overall knowledge score was a significant predictor of BSE performance. This was in agreement with the results of other previous studies [61–63]. It has been reported that increased breast awareness may have contributed in the decrease in mortality from breast cancer in some countries, although uncertainty exists whether the benefits of BA outweigh the harms [16]. Thus, involvement of the women in the community to participate in the development and implementation of health education programs on breast awareness is a necessity [61].

Madanat and Merrill [64] have reported that women with a history of breast cancer in the family have more general information on breast cancer and awareness of breast cancer screening tests than other women. Studies have also reported that women with a history of breast cancer in the family perform BSE more regularly [58, 65]. This was in agreement with the present study where family history of BC was a significant predictor of BSE performance. The reason may be that they know the relationship between genetic factors and breast cancer, so they see themselves as possessing such risk factors. One of the reasons preventing women from performing screening methods such as BSE regularly was the fear of finding a mass during the examination and the fear of surgery [5, 66, 67].

This study has some limitations. First, because the study was cross-sectional, a temporal relationship between exposure and outcome cannot be established. It is clear that the true causal relationships among all of the identified variables are complex and often reciprocal. For example, knowledge and attitude of the women on BSE may be affected by many confounders other than those used in the present study. Second, the small number of. Second, it was conducted in one center, and women who participated in the study may not be representative of the whole female population in Saudi Arabia, and this would affect the generalizability of the results. Another limitation is that the data collection was based on a self administered questionnaire, thus data might have been subjected to information bias (recall bias).

## Conclusion

In the present study, women had poor knowledge on breast cancer, reported negative attitude towards BSE and their practice was poor. Reasons for not doing BSE as reported not knowing how to examine their breast was the main reason for poor practice. Women with higher perception of BSE confidence have lower risks for not doing BSE, and women having lower levels of BSE barriers have higher potential for doing BSE. Employment was the only significant socio-demographic predictor of BSE practice. The role of health providers was limited, reflecting the need to improve awareness programs by health care professionals. These findings suggest that there is a need for continuing education programs to upgrade knowledge, change attitude, confidence and behavior towards BSE. Emphasis should be laid on BSE in undergraduate and postgraduate courses. It is important to establish specialized resource centers in different regions in Saudi Arabia, to promote and integrate BSE training programs for all working women. Periodic follow up of female employees and other women in different settings in the community is very important to ensure early detection of cases. Meanwhile, BSE training programs must be adopted as one of the routine services offered to the working females. Health professionals should advise women to be “breast aware” and inform them what changes may indicate cancer and how to seek appropriate advice.

## Abbreviations

BC: Breast cancer
BSE: Breast self examination
HBM: Health Belief Model
CHBMS: Champion’s Health Belief Model Scale
CAM: Cancer Awareness Measure
PMS: Percentage mean score
CBE: Clinical breast examination
BA: Breast awareness
KAMC: King Abdulaziz Medical City
HRT: Hormone replacement therapy
OCP: Oral contraceptive pills
KAIMRC: King Abdullah International Medical Research Center
